# Integrated Analysis of lncRNA and circRNA Mediated ceRNA Regulatory Networks in Skin Reveals Innate Immunity Differences Between Wild-Type and Yellow Mutant Rainbow Trout (*Oncorhynchus mykiss*)

**DOI:** 10.3389/fimmu.2022.802731

**Published:** 2022-05-17

**Authors:** Shenji Wu, Jinqiang Huang, Yongjuan Li, Zhe Liu, Lu Zhao

**Affiliations:** ^1^ College of Animal Science and Technology, Gansu Agricultural University, Lanzhou, China; ^2^ College of Science, Gansu Agricultural University, Lanzhou, China

**Keywords:** rainbow trout, innate immunity, whole transcriptome sequencing, lncRNA-miRNA-mRNA network, circRNA-miRNA-mRNA network

## Abstract

Fish skin is a vital immune organ that forms the first protective barrier preventing entry of external pathogens. Rainbow trout is an important aquaculture fish species that is farmed worldwide. However, our knowledge of innate immunity differences between wild-type (WR_S) and yellow mutant rainbow trout (YR_S) remains limited. In this study, we performed whole transcriptome analysis of skin from WR_S and YR_S cultured in a natural flowing water pond. A total of 2448 mRNAs, 1630 lncRNAs, 22 circRNAs and 50 miRNAs were found to be differentially expressed (DE). Among these DEmRNAs, numerous key immune-related genes, including *ifih1*, *dhx58*, *trim25*, *atp6v1e1*, *tap1*, *tap2*, *cd209*, *hsp90a.1*, *nlrp3*, *nlrc3*, and several other genes associated with metabolism (*gstp1*, *nampt*, *naprt* and *cd38*) were identified. Gene Ontology (GO) and Kyoto Encyclopedia of Genes and Genomes (KEGG) pathway enrichment analyses of DEmRNAs revealed that many were significantly enriched in innate immune-related GO terms and pathways, including NAD+ADP-ribosyltransferase activity, complement binding, immune response and response to bacterium GO terms, and RIG-I-like receptor signaling, NOD-like receptor signaling and phagosome KEGG pathways. Furthermore, the immune-related competing endogenous RNA networks were constructed, from which we found that lncRNAs MSTRG.11484.2, MSTRG.32014.1 and MSTRG.29012.1 regulated at least three immune-related genes (*ifih1*, *dhx58* and *irf3*) through PC-5p-43254_34, PC-3p-28352_70 and bta-miR-11987_L-1R-1_1ss8TA, and *tap2* was regulated by two circRNAs (circRNA5279 and circRNA5277) by oni-mir-124a-2-p5_1ss13GA. The findings expand our understanding of the innate immune system of rainbow trout, and lay the foundation for further study of immune mechanisms and disease resistance breeding.

## Introduction

Compared with terrestrial vertebrates, fish inhabit a comparatively complicated aquatic environment containing a wide variety of pathogenic microorganisms, and they are regularly exposed to adverse environmental changes (1). Fish skin is the first line of defence against these hazards, protecting the organism from its environment and hindering the entry of pathogens (2). In addition to being a physical protective barrier, it also performs biological functions such as thermal regulation and metabolic activity (3, 4), which are important for maintaining homeostasis and supporting the normal physiological functions of fish. Unlike mammalian skin, the fish epidermis is attached with mucin-enriched mucus generated mainly by goblet cells, and contains living epithelial cells that make direct contact with the surrounding aquatic environment (5, 6). In addition to secretory cells, fish skin contains active immune sites harbouring cellular defences including leukocytes (granulocytes, macrophages and lymphocytes) and dendritic-like cells (7, 8). Moreover, six types of pigment cells (melanocytes, xanthophores, erythrophores, iridophores, leucophores and cyanophores) and an intricate microbiome including commensals and pathogens have been identified in fish skin (6, 9). Previous study in black-boned chicken (*Gallus domesticus*) demonstrated that melanocytes were kind of immune cells that exerted important innate immune roles during infectious bursal disease virus infection (10). In zebrafish (*Danio rerio*), the melanocytes could engulf exogenous bead and then recruit immune cells to protect from injury (11). These examples illustrated the number of skin melanocytes is closely correlated with mucosal immunity. Thus, the skin mucosal immune system is clearly complicated.

So far, lots of fish skin transcription profiles for various species have been obtained including orange-spotted grouper (*Epinephelus coioides*) (12), rabbitfish (*Siganus oramin*) (13), yellow croaker (*Larimichthys crocea*) (14), zebrafish (15) and rainbow trout (6). These studies showed that numerous immune-related genes were upregulated upon infection with various pathogens, as is the case for Toll-like receptors (*tlrs*), NOD-like receptors (*nlrs*), janus kinases (*jaks*), transporter (*tap*), signal transducer and activator of transcriptions (*stats*) and interferon regulatory factor 3 (*irf3*), implying that resistance to stress is proportional to the expression levels of certain immune-related genes. Furthermore, fish skin is rich in B- and T-cells as well as serves as a repository of many innate immune components, such as immunoglobulins (IgM, IgD and IgT), lysozyme, lectins, antimicrobial peptides and C-reactive protein, of which IgT is thought to be specialized in mucosal immunity (16–18). In rainbow trout, IgT protein concentration and IgT^+^ B-cells numbers significantly increase in skin mucus following *Ichthyophthirius multifiliis* challenge (18, 19). The above results suggested that fish skin plays an essential role in defending against pathogens.

Interestingly, in addition to the protein-coding RNAs, accumulating evidence has revealed non-coding RNAs (ncRNAs) also exert vital effects on a remarkable variety of biological processes, especially in immunity (20). MicroRNAs (miRNAs) are common ncRNAs involved in regulating immune response, which can repress gene expression by inhibiting mRNA translation or promoting mRNA degradation (21). Recently, two other types of ncRNA, long non-coding RNAs (lncRNAs) and circular RNAs (circRNAs) were discovered in vertebrate. LncRNAs are RNA molecules longer than 200 nucleotides and can regulate gene expression *via* cis/trans-acting or miRNA sponges (22). CircRNAs are covalently closed circular molecule generated by head to-tail splicing at the splice sites (23). According to the theory of competing endogenous RNA (ceRNA), lncRNA and circRNA can act as ceRNAs through competitively binding common miRNA response elements (MREs), and forming complex miRNA-mediated ceRNA networks, resulting in suppression of miRNAs and the expression of corresponding target genes (24). In recent years, ceRNA has provided a new way to study immune mechanisms of fish, for instance, a study in miiuy croaker (*Miichthys miiuy*) reported that lncRNA NARL exhibits a positive regulatory role in inflammatory and antiviral responses *via* acting as a ceRNA for miR-217-5p to relieve its repressive effects on nucleotide-binding oligomerization domain containing 1 (*nod1*) expression (25). Similarly, circRNA circDTx1, a ceRNA of Toll–interleukin 1 receptor domain-containing adaptor molecule (*trif*), was involved in anti-*Siniperca chuatsi rhabdovirus* response by sponging miR-15a-5p, resulting in activation of the NF-κB/IRF3 pathway (26). Under normal conditions fish maintain a healthy status by defending themselves against potential invaders using a repertoire of innate and specific defense mechanisms (27). However, prior researches mainly focused on identification and characterization of ceRNAs and ceRNA regulatory networks in fish spleen, liver, and intestine following artificial infection to explore the immune mechanisms of pathogen resistance (24, 28–30), and no study on the involvement of ceRNA in fish skin without artificial infection has been documented. Owing to many biological and abiotic stresses presented in natural flowing water pond aquaculture environment, and intensified culture environment and high susceptibility to infectious disease make the industry vulnerable to disease outbreaks resulting in significant economic losses. Therefore, studies on fish skin without artificial infection are necessary.

Rainbow trout is an economically important cold water fish, and it has been widely cultivated worldwide. This species contributes to a large proportion of freshwater aquaculture production. Two phenotypes of rainbow trout are most common in farms; wild-type rainbow trout with black skin (WR_S) and yellow mutant rainbow trout with yellow skin (YR_S). Differences in skin colour are caused by differences in the composition of pigment cells. In WR_S, melanocytes and xanthophores are present, whereas melanocytes are absent in YR_S, and there are fewer xanthophores than in WR_S (31). Furthermore, survival is lower among YR_S variants than WR_S (32). We hypothesized that differences in survival may be caused by differences in immunity between these two strains. Fish skin is an important immune organ, however, little is known about the differences in immunity between WR_S and YR_S in a natural flowing water pond aquaculture environment, and very few studies were conducted to investigate the ceRNA mechanism for fish skin. In the present study, whole transcriptome sequencing was performed to uncover global molecular expression differences of skin between WR_S and YR_S at both mRNAs and ncRNAs levels. We identified numerous differentially expressed (DE) mRNAs, DElncRNAs, DEcircRNAs and DEmiRNAs, and characterized the immune-related ceRNA networks of lncRNA-miRNA-mRNA and circRNA-miRNA-mRNA. These new ceRNA networks assist with developing a better understanding of innate immune mechanisms, and provide theoretical guidance for disease resistance breeding in rainbow trout.

## Materials and Methods

### Experimental Animals

**Figure 1:**
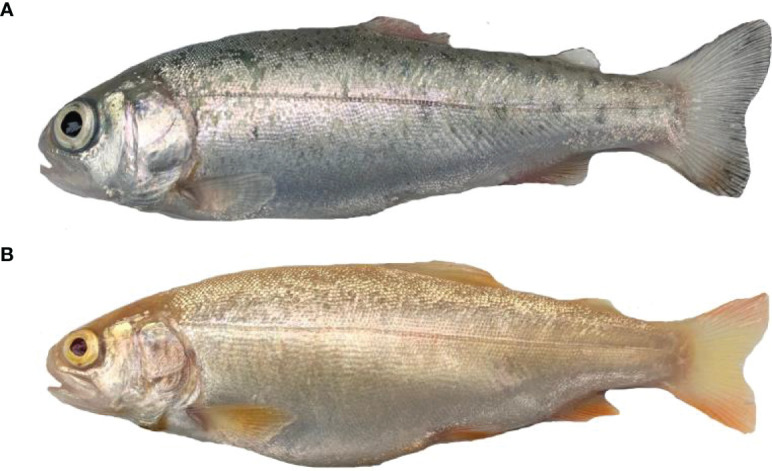
Two phenotypes of rainbow trout. **(A)** Wild-type rainbow trout (WR_S). **(B)** Yellow mutant rainbow trout (YR_S).

Female WR_S and YR_S (160 ± 3.5 g) used in this study were obtained from a fish farm in Gansu Province, China, and all these fish were cultured in the same natural flowing water pond (20000 L) at 13 ± 0.1°C with 8.5 ± 0.1 mg/L dissolved oxygen, pH 7.2 ± 0.1 and ammonia nitrogen concentration of less than 0.05 mg/L (**Figure 1**). All fish were fed a commercial pellet feed (~6% of body weight) at 9 am and 3 pm every day. Culture conditions were as stated by the Standard of Linxia Salmon and Trout Elite Breeding and Protection Farm (Gansu, China; approved by the Department of Agriculture, China, 2009). Three dorsal skins of WR_S (WR_S1, WR_S2, WR_S3) and three dorsal skins of YR_S (YR_S1, YR_S2, YR_S3) with similar size and body colour were randomly selected for RNA-seq. These fish were anesthetised with a lethal dose of MS-222 (400 mg/L) before tissue sampling, dorsal skin samples (4.5–5 cm^2^) were immediately collected and flash-frozen in liquid nitrogen, and stored at −80°C. All experiments complied with institutional guidelines and the protocol approved by the Animal Experimentation Ethics Committee at Gansu Agricultural University, China.

### RNA Extraction, Library Construction and Sequencing

Total RNA was extracted from six samples using TRIzol reagent (Invitrogen, Carlsbad, CA, USA) according to the manufacturer’s procedure. The purity, quantity and integrity (28S/18S) of total RNA were examined by 1.5% agarose gel electrophoresis stained with Goldview nucleic acid dye (Solarbio, Beijing, China), NanoDrop ND-1000 instrument (NanoDrop, Wilmington, DE, USA) and Agilent 2100 Bioanalyzer (Agilent Technologies, Palo Alto, CA, USA). Approximately 1 µg of total RNA with an RNA integrity number (RIN) ≥ 7 was selected for small RNA library construction using a TruSeq small RNA Sample Prep Kit (Illumina, San Diego, CA, USA) following the manufacturer’s protocol. Then six libraries (three WR_S and three YR_S) were sequenced by Illumina Hiseq 2500, and 50 bp single-end reads were generated.

For mRNA, lncRNA and circRNA sequencing, about 5 μg of total RNA from each sample was used to deplete ribosomal RNA using the Ribo-Zero rRNA Removal Kit (Illumina, San Diego, USA) according to the manufacturer’s instruction. After removing ribosomal RNAs, RNAs were fragmented by divalent cations under a high temperature and reverse transcribed into cDNA, which was used to synthesize U-labeled second-stranded DNAs with DNA polymerase I, RNase H and dUTP and buffer. Finally, 150 bp paired-end reads were generated by an Illumina Hiseq 4000 platform based on the paired-end sequencing.

### Identification of mRNA, lncRNA, circRNA and miRNA

Clean data were generated by eliminating reads containing adaptor contamination, poly-N sequences, and low-quality reads using cutadapt. Then the sequence quality of remaining reads was validated by FastQC (http://www.bioinformatics.babraham.ac.uk/projects/fastqc/). Clean reads were aligned to the rainbow trout reference genome (https://www.genoscope.cns.fr/trout/) by Bowtie2 and Tophat2 with the default parameters (33). The mapped reads of each sample were assembled using StringTie, and a comprehensive transcriptome was reconstructed through merging all the samples. StringTie and Ballgown were used to estimate the expression profiles of all the transcripts (34).

For identification of novel lncRNAs, the transcripts that overlapped with known mRNAs and transcripts shorter than 200 bp were filtered (35). We used Coding Potential Calculator (CPC), Coding-Non-Coding-Index (CNCI) and Pfam to predict transcripts with coding potential (22). According to the prediction results, the transcripts with CPC score < −1 and CNCI score < 0 were discarded and those remaining were considered as lncRNAs.

According to the pipeline used in the analysis of mRNA discovery, the remaining reads (unmapped reads) were still mapped to genome to find unique anchor positions within the splice site using Tophat-fusion (36). CIRCExplorer2 (37) and CIRI (38) tools were used to identify circRNAs in this study. In view of the high false positives in circRNA identification, the overlapped outputs from CIRCExplorer2 and CIRI were kept for further analysis.

Raw reads generated from miRNA sequencing libraries were subjected to ACGT101-miR (LC Sciences, Huston, TX, United States) to filter out adapter dimers, junk, low complexity, repeats, rRNA, tRNA, snRNA, and snoRNA. All of the clean tags were searched against the miRBase database (Release 22) to identify known miRNAs in rainbow trout. All of the unannotated tags were analysed by the position of their genome and hairpin structures by Mireap_v0.2 software, and the novel miRNA candidates were identified.

### Screening of DEmRNAs, DElncRNAs, DEcircRNAs and DEmiRNAs, and Gene Function Annotation

As reported previously (39), the DESeq2 (v.1.6.3) in R was used to conduct differential expression analysis between WR_S and YR_S groups (40). mRNAs and lncRNAs with |log2 fold change| ≥ 1 and *q*value < 0.05 were identified as significantly DEmRNAs and DElncRNAs. Additionally, significantly DEcircRNAs and DEmiRNAs were identified with a |log2 fold change| ≥ 1 and *p*value < 0.05. To further evaluate the biological functions and potential mechanisms of mRNAs, DEmRNAs were then subjected to an enrichment analysis of GO and KEGG pathways. GO enrichment and KEGG pathway analyses were performed using the GOseq R package (Release 2.12) and the KOBAS software (v2.0), respectively. The statistical significance was examined using the hypergeometric test, and *q*value < 0.05 was considered significant for GO and KEGG enrichment. Additionally, a protein-protein interaction (PPI) network of DEmRNAs was constructed using the Search Tool for the Retrieval of Interacting Genes/Proteins (STRING, version 10.0) database.

### Construction and Analysis of ceRNAs Regulatory Network

To reveal the roles and interactions of DEmRNAs, DElncRNAs, DEcircRNAs and DEmiRNAs, two ceRNA networks were constructed based on the theory of ceRNA, i.e., the lncRNA-miRNA-mRNA and circRNA-miRNA-mRNA networks. We paid more attention to the positive correlations expression of lncRNA-mRNA and circRNA-mRNA, so the miRNAs capable of simultaneously regulating lncRNA/circRNA and mRNA were focused on. Targets of miRNAs were predicted to construct the lncRNA/circRNA-miRNA-mRNA networks. The pairwise corrections of lncRNA/circRNA-miRNA and miRNA-mRNA were evaluated using miRanda 3.3a and TargetScan 5.0 (33). The analysis and visualization of the interactions were performed by Cytoscape software (v3.6.0).

### qRT-PCR Validation

qRT-PCR was performed to confirm the expression levels of DEmRNAs DElncRNAs, DEcircRNAs and DEmiRNAs from RNA-seq. The RNA used for Illumina sequencing was also used here for the validation. Total RNA was subjected to cDNA synthesis using a PrimerScript RT Reagent Kit with gDNA Eraser (TaKaRa, Dalian, China) and a Mir-X miRNA First-Strand Synthesis Kit (Clontech, Mountain View, CA, USA) following the manufacturer’s instructions. For lncRNA, circRNA and mRNA quantification, the 20 μL reaction volume contained 10 μL of SYBR Premix Ex Taq II (2×), 1 μL of each the sense and antisense primer (10 μM), 0.5 μL of cDNA, 7.5 μL ddH_2_O. The mRNA, lncRNA and circRNA expression levels were normalized to *β-actin* (24). For miRNA quantification, the 20 μL reaction volume contained 10 μL of SYBR Premix Ex Taq II (2×), 0.4 μL of each sense and antisense primer (10 μM), 1.6 μL of cDNA, 7.6 μL of ddH_2_O. The miRNA expression level was normalized to U6 (41). PCR amplification procedure for all experiments were carried out at 95°C for 30 s, followed by 40 cycles at 95°C for 5 s and 60°C for 30 s. Target specificity was determined by melting curve analysis, and RNA expression levels of target genes versus *β-actin* or U6 were calculated using the 2^-ΔΔCt^ method. All results are expressed as means ± SD, and statistical analyses were performed using one-way ANOVA followed by Dunn’s test in SPSS (version 22.0) (42), and Pearson correlation analysis was used to calculate the correlation value between qRT-PCR and RNA-seq results. All primers are included in **Table S1**.

## Results

### Overview of RNA-Sequencing Results

A total of 583,011,930 raw reads were generated in strand-specific library using an Illumina Hiseq 4000 system, and were deposited at the National Center for Biotechnology Information (NCBI) database under accession numbers GSE153997. After discarding low-quality sequencing data, 557,976,332 clean reads were obtained. The average of quality Q20 and Q30 were respectively higher than 99.35% and 93.53% for each library, and the average GC content was 48.42%. Additionally, an average of 84.87% (55.97% unique mapped reads and 28.90% multi mapped reads) of the clean reads per sample was mapped to the rainbow trout genome (**Table S2**). Single sequencing of the miRNA library yielded a total of 82,053,134 raw reads, which were submitted to the NCBI database under the accession number GSE181974. After discarding junk sequences, 38,307,832 and 31,927,956 clean reads were obtained from WR_S and YR_S groups, respectively. (**Table S3**).

### Identification of DEmRNAs, DElncRNAs, DEcircRNAs and DEmiRNAs Between WR_S and YR_S

**Figure 2:**
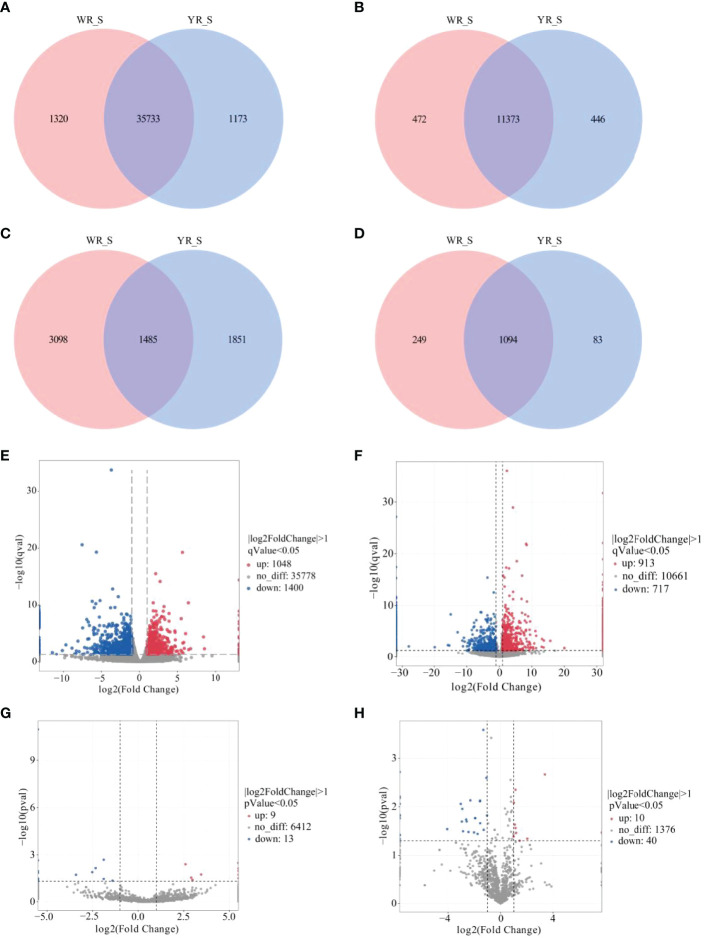
Comparative analysis of mRNA and ncRNAs between WR_S and YR_S groups. **(A)** mRNAs, **(B)** lncRNAs, **(C)** circRNAs and **(D)** miRNAs expressed only in WR_S (pink circle), only in YR_S (blue circle), and co-expressed in both WR_S and YR_S (intersection). **(E)** Volcano plot of differentially expressed DE mRNAs. **(F)** Volcano plot of DElncRNAs. **(G)** Volcano plot of DEcircRNAs. **(H)** Volcano plot of DEmiRNAs.

**Figure 3:**
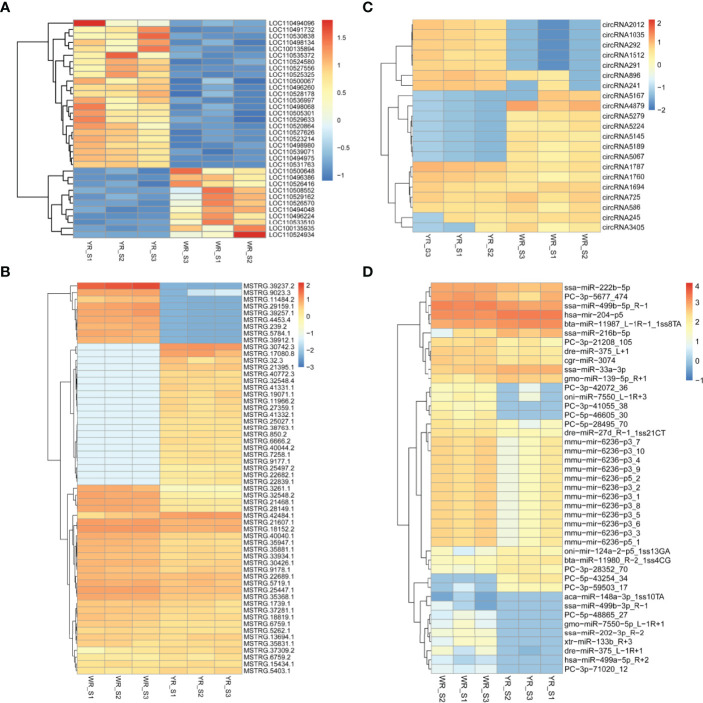
Unsupervised clustering analysis of DEmRNAs and DEncRNAs between WR_S and YR_S groups. **(A)** DEmRNAs. **(B)** DElncRNAs. **(C)** DEcircRNAs. **(D)** DEmiRNAs.

A total of 38,226 genes were identified from comparison of WR_S and YR_S groups, and 35,733 genes were co-expressed in both groups, while 1320 genes were only expressed in WR_S and 1173 genes were only expressed in YR_S (**Figure 2A**). With fold-change in expression ≥ 2 and *q*value < 0.05 as thresholds, 2448 DEmRNAs were identified, among which 1048 were upregulated and 1400 were downregulated in YR_S group (**Figure 2E**). In addition, 12,291 lncRNAs, 6434 circRNAs and 1426 miRNAs were obtained, of these, 472 lncRNAs, 3098 circRNAs and 249 miRNAs were only expressed in WR_S and 446 lncRNAs, 1851 circRNAs and 83 miRNAs were only expressed in YR_S (**Figures 2B–D**). Compared with WR_S group, these were 1630 lncRNAs (913 up- and 717 downregulated), 22 circRNAs (9 up- and 13 downregulated) and 50 miRNAs (10 up- and 40 downregulated) that were differentially expressed in YR_S group (**Figures 2F–H** and **Table S4**). Besides, the general expression profiles of DEmRNAs, DElncRNAs, DEcircRNAs and DEmiRNAs from the six simples were analysed by hierarchical clustering (**Figures 3A–D**).

### Sequence Characterization of mRNAs and ncRNAs

Comparison of the transcriptome characterizations between lncRNA and mRNA showed that more than 80% of the mRNAs was greater than 1000 bp long, whereas more than 60% of the lncRNAs was less than 1000 bp long (**Figure S1A**); for exon number, majority of lncRNAs were concentrated at one to three exons that was not the case for mRNAs, which contained a higher fragments per kilobase of transcripts per million fragments mapped (FPKM) value and longer ORF length (**Figures S1B–D**). Besides, the percentages of lncRNAs, sense lncRNAs, antisense lncRNAs and intronic lncRNAs were 44.87, 18.56, 14.70 and 21.87%, respectively (**Figure S1E**). For circRNAs, the sequence length distribution of circRNAs is shown in **Figure S1F**, and most of them were 200 bp to 600 bp, or longer than 1000 bp. Additionally, the majority of circRNAs belong to the exon type (77.69%) (**Figure S1G**). For miRNAs, the length distributions of miRNAs showed that most of the miRNAs were 21–23 nt long, and the 22 nt miRNAs were most abundant, representing 36.94% and 37.54% of the miRNAs in WR_S and YR_S groups (**Figure S1H**).

### GO Enrichment and KEGG Pathway Analyses of DEmRNAs

**Figure 4:**
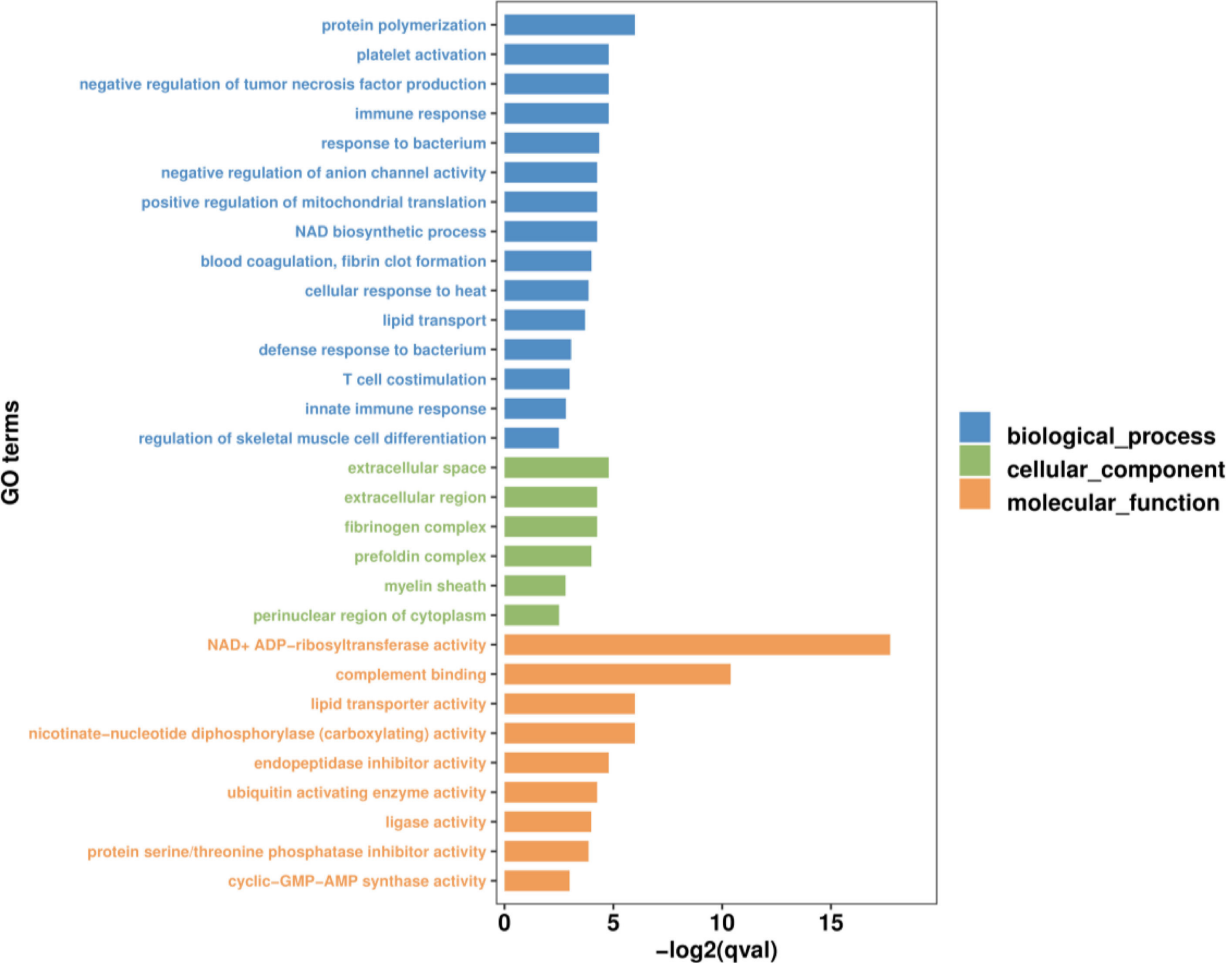
The top 30 most significant level-3 GO terms of DEmRNAs between WR_S and YR_S groups. The x-axis represents −log2 (*q*value), the y-axis represents GO_terms.

GO and KEGG functional enrichment analyses were performed to explore the biological and functional roles of the identified DEmRNAs. GO functional enrichment analysis of DEmRNAs was divided into three categories: biological process, cellular component and molecular function. With *q*value < 0.05 as the threshold, five sub-categories belonging to the ‘biological processes’ category were identified; ‘protein polymerization’ (GO:0051258), ‘immune response’ (GO:0006955), ‘negative regulation of tumor necrosis factor production’ (GO:0032720), ‘platelet activation’ (GO:0030168) and ‘response to bacterium’ (GO:0009617). Five sub-categories belonging to the ‘molecular function’ category were identified; ‘NAD+ADP-ribosyltransferase activity’ (GO:0003950), ‘complement binding’ (GO:0001848), ‘nicotinate-nucleotide diphosphorylase (carboxylating) activity’ (GO:0004514), ‘lipid transporter activity’ (GO:0005319) and ‘endopeptidase inhibitor activity’ (GO:0004866). One sub-categories belonging to the ‘cellular component’ category was identified; ‘extracellular space’ (GO:0005615). The top 30 most significant level-3 GO terms in these three categories, and accompanying detailed information, are presented in **Figure 4** and **Table S5**, respectively.

**Figure 5:**
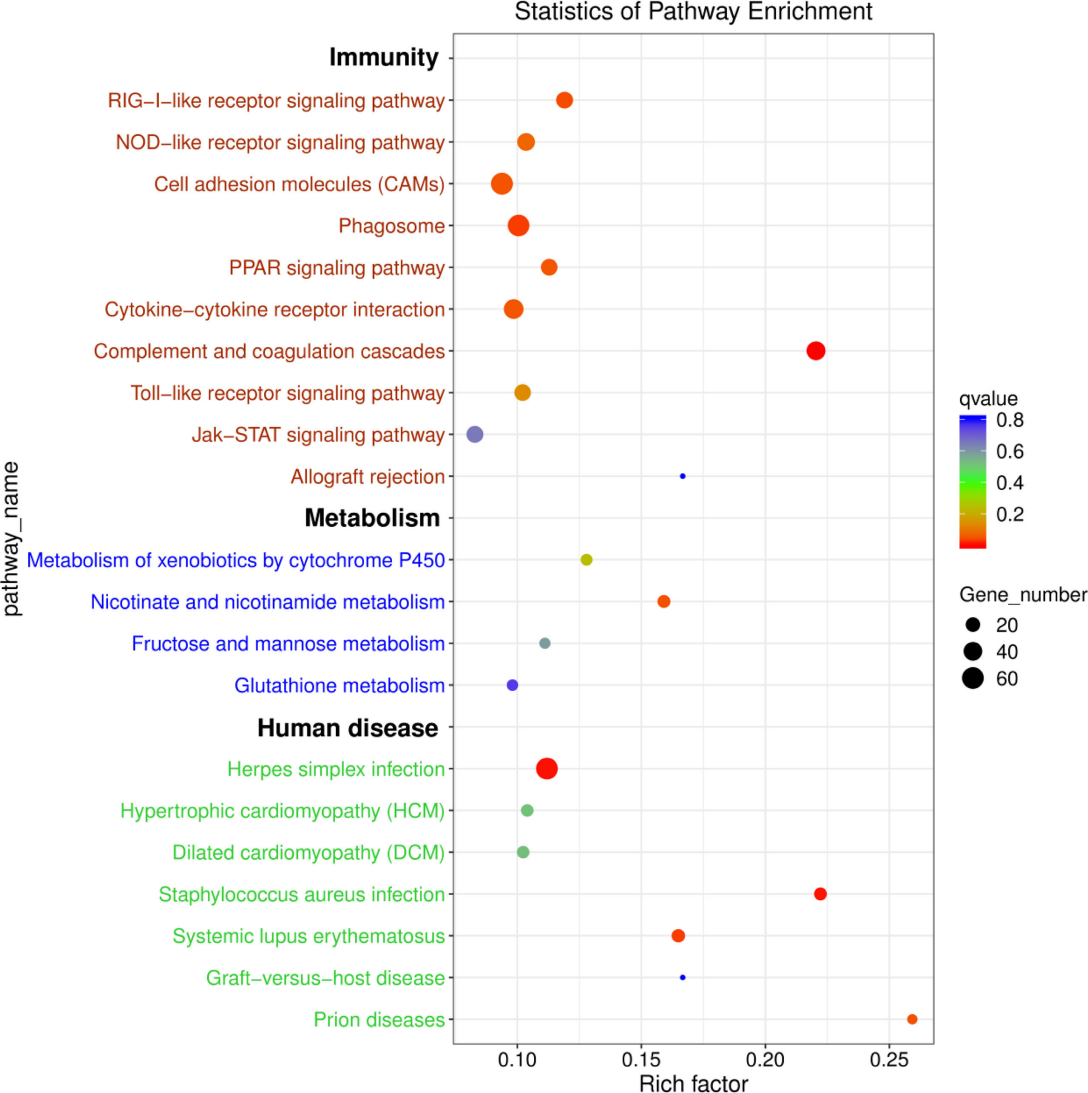
KEGG enrichment diagram of DEmRNAs between WR_S and YR_S groups. The x-axis represents the rich factor, the y-axis represents different pathways. Different colours of plots indicate different *q*values. The plot diameter represents the number of DEmRNAs in a pathway.

**Figure 6:**
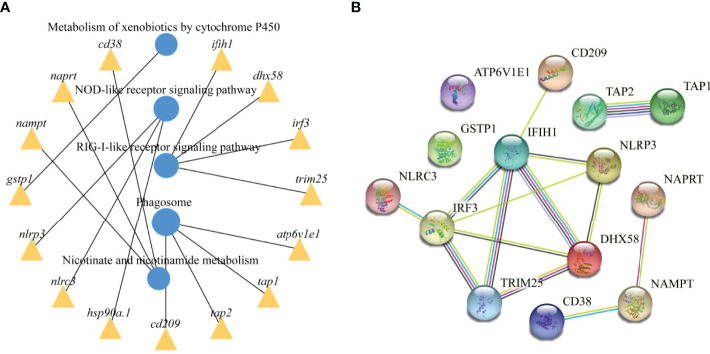
Immune and metabolism-related pathways and genes interactions. **(A)** Enrichment of DEmRNAs in corresponding pathways and interaction of immune and metabolism-related pathways. **(B)** PPI network analysis of the immune and metabolism-related genes identified in this study based on STRING database.

To further explore the functions of the identified DEmRNAs, we analysed the transcriptome results using the KEGG database. As shown in **Figure 5**, dozens of pathways involved in immunity, metabolism and human disease were identified. A total of nine pathways were enriched with *q*value < 0.05, including complement and coagulation cascades, herpes simplex infection, *Staphylococcus aureus* infection, systemic lupus erythematosus, phagosome, RIG-I-like receptor signaling pathway, prion diseases, fat digestion and absorption, nicotinate and nicotinamide metabolism (**Table S6**). Furthermore, numerous key DEmRNAs were found to be related to immune system and metabolism, including interferon induced with helicase C domain 1 (*ifih1*), DEXH (Asp-Glu-X-His) box polypeptide 58 (*dhx58*), *irf3*, tripartite motif containing 25 (*trim25*), ATPase H^+^ transporting V1 subunit E1 (*atp6v1e1*), *tap1*, *tap2*, CD209 antigen (*cd209*), heat shock protein 90, class A member 1 (*hsp90a.1*), NLR family, CARD domain containing 3 (*nlrc3*), NLR family pyrin domain containing 3 (*nlrp3*), glutathione S-transferase pi 1 (*gstp1*), nicotinamide phosphoribosyltransferase (*nampt*), nicotinate phosphoribosyltransferase (*naprt*) and cyclic ADP-ribose hydrolase 1 (*cd38*), and most of them were upregulated in WR_S. Detailed information is included in **Table 1**. Additionally, interactions of immune and metabolism-related pathways and genes are shown in **Figure 6**.

**Table 1:** Representative immune and metabolism-related genes differentially expressed between wild-type rainbow trout (WR_S) and yellow mutant rainbow trout (YR_S) groups.

Gene name	Note	YR_S FPKM	WR_S FPKM	Log2 (FC)	*q*value
**Immune-related**					
*ifih1*	Melanoma differentiation associated gene 5	7.12	43.04	-2.60	0.02
*dhx58*	DExH-box helicase 58	4.68	57.78	-3.63	4.11×10^-3^
*irf3*	Interferon regulatory factor 3	19.52	219.04	-3.94	0.01
*trim25*	E3 ubiquitin	0.59	4.88	-3.06	9.70-06
*atp6v1e1*	V-type proton ATPase subunit E 1	14.22	4.52	1.65	1.69×10^-3^
*tap1*	Antigen peptide transporter 1	6.86	30.28	-2.14	0.02
*tap2*	Antigen peptide transporter 2	3.02	21.42	-2.83	0.02
*cd209*	CD209 antigen	13.92	159.35	-3.52	2.09×10^-4^
*hsp90a.1*	Heat shock protein HSP 90-alpha 1	2.25	36.40	-4.01	3.61-07
*nlrc3*	NLR family CARD 3	0.45	3.86	-3.12	1.69×10^-3^
*nlrp3*	NACHT, LRR and PYD domains 3	1.93	9.39	-2.28	0.02
**Metabolism related**					
*gstp1*	Glutathione S-transferase P1	21.06	6.22	1.76	6.61×10^-4^
*nampt*	Nicotinamide phosphoribosyltransferase	3.50	37.74	-3.43	5.94×10^-4^
*naprt*	Nicotinate phosphoribosyltransferase	0.19	1.04	-2.48	1.72×10^-3^
*cd38*	Cyclic ADP-ribose hydrolase 1	0.82	6.18	-2.91	0.02

### ceRNA Regulatory Networks Construction

**Figure 7:**
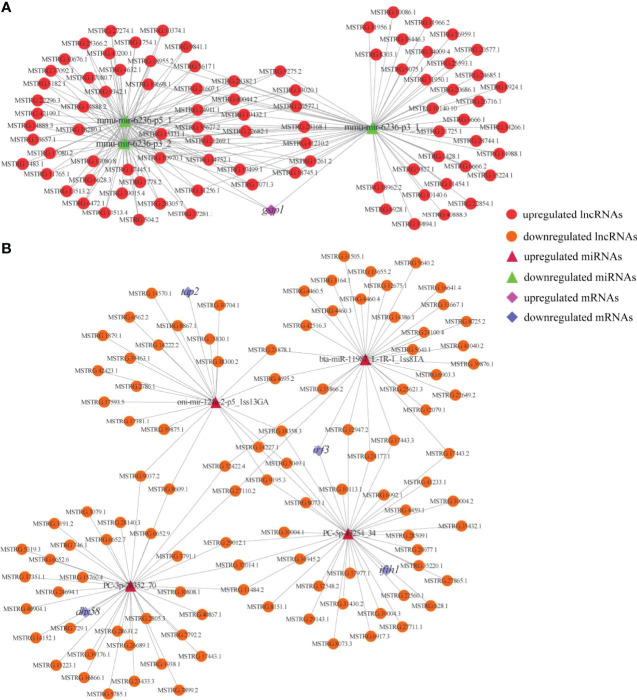
Immune and metabolism-related DElncRNA-DEmiRNA-DEmRNA ceRNA networks. **(A)** A ceRNA network of metabolism-related gene. **(B)** A ceRNA network of immune-related genes. The red circles, green triangles and pink diamonds represent upregulated DElncRNAs, downregulated DEmiRNAs and upregulated DEmRNA, respectively. The yellow circles, wine red triangles and blue diamonds represent downregulated DElncRNAs, upregulated DEmiRNAs and downregulated DEmRNAs, respectively.

**Figure 8:**
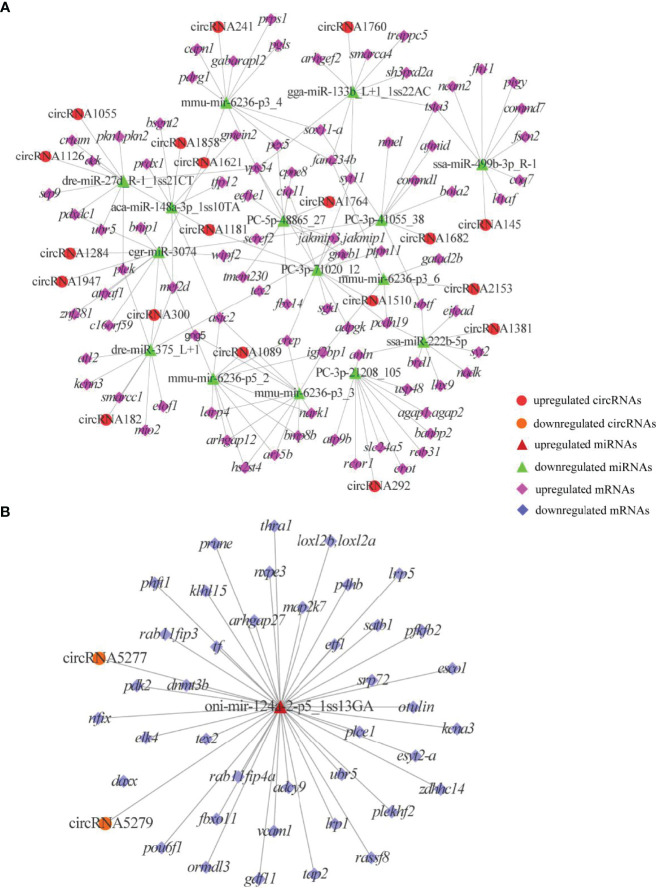
DEcircRNAs-mediated ceRNA networks. **(A)** A ceRNA network of upregulated genes. **(B)** A ceRNA network of downregulated genes. The red circles, green triangles and pink diamonds represent upregulated DEcircRNAs, downregulated DEmiRNAs and upregulated DEmRNAs, respectively. The yellow circles, wine red triangles and blue diamonds represent downregulated DEcircRNAs, upregulated DEmiRNAs and downregulated DEmRNAs, respectively.

To reveal the global regulatory network of immune-related mRNAs and ncRNAs, two ceRNA regulatory networks were constructed using DEmRNAs, DElncRNAs, DEcircRNAs and DEmiRNAs based on ceRNA theory. By using lncRNA as a decoy, miRNA as the center, and mRNA as the target, 326 lncRNA-miRNA-mRNA interactions were finally obtained, including 88 upregualted and 106 downregulated lncRNAs, 4 upregulated and 3 downregulated miRNAs, 1 upregulated (*gstp1*) and 4 downregulated mRNAs (*ifih1*, *dhx58*, *irf3* and *tap2*) (**Figure 7** and **Table S7**). By using circRNA as a decoy, miRNA as the center, and mRNA as the target, resulting in finally 3061 interaction relationships of circRNA-miRNA-mRNA. There are 20 upregulated and 2 downregulated circRNAs, 1 upregulated and 17 downregulated miRNAs, and 963 upregulated and 177 downregulated mRNAs. It is worth mentioning that two circRNAs (circRNA5277 and circRNA5279) were predicted to interact with *tap2* through oni-mir-124a-2-p5_1ss13GA. The circRNA-miRNA-mRNA network was shown in **Figure 8** and **Table S8**.

### Validation of RNA-Seq Data by qRT-PCR

**Figure 9:**
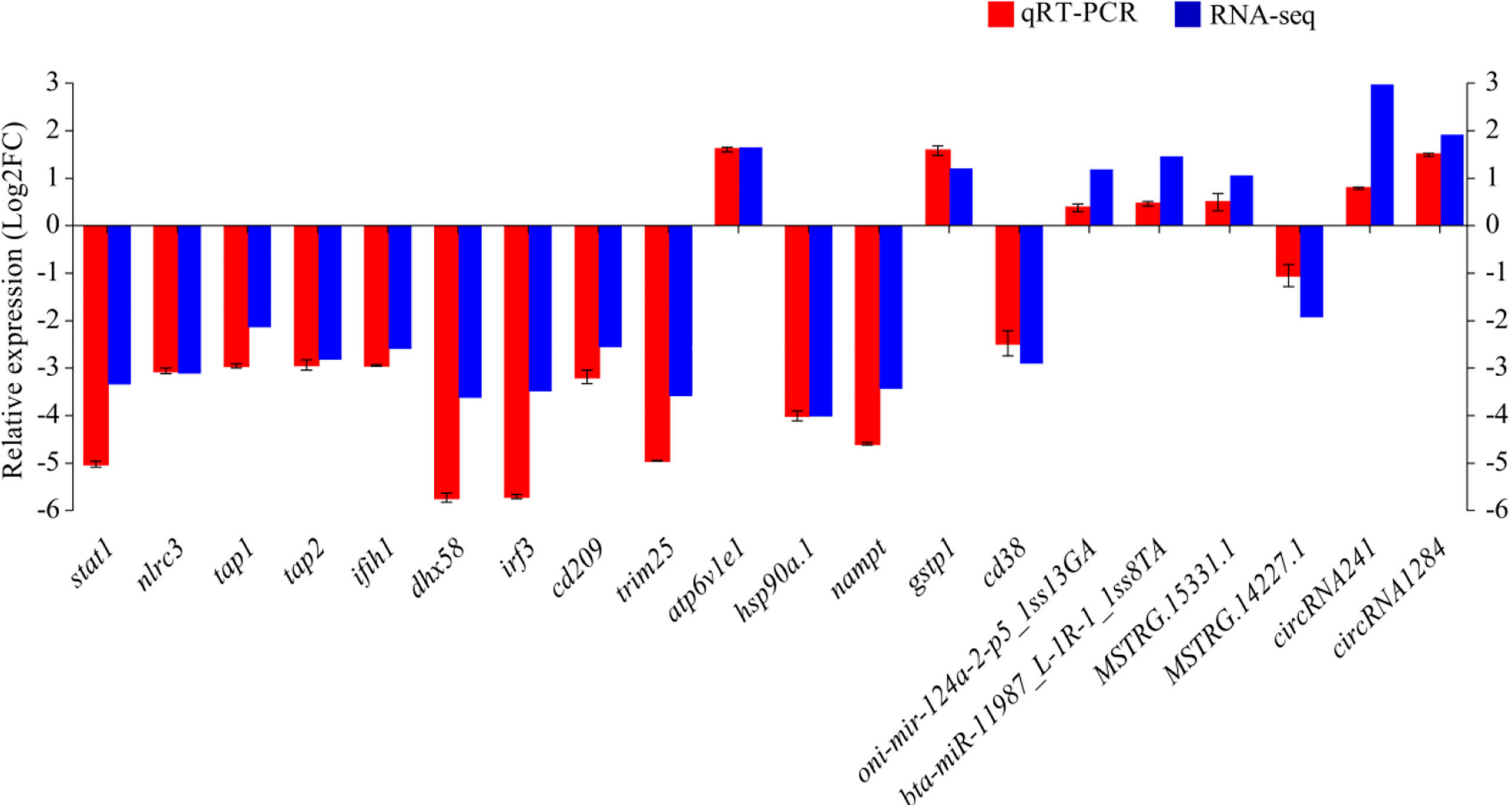
The differential expression of mRNAs and ncRNAs in RNA-seq was validated by qRT-PCR. Fold-change of qRT-PCR data represents the ratio of target gene expression values for YR_S vs. WR_S after normalization against *β-actin*/U6.

qRT-PCR was performed on 14 DEmRNAs and six DEncRNAs to validate the expression patterns that were identified by RNA-seq. The results showed that qRT-PCR expression patterns were consistent with the RNA-seq data (**Figure 9**), and the statistical analysis showed correlation of R = 0.938 between the two types of analysis, confirming the reliability of the RNA-seq data.

## Discussion

Fish skin, considered the largest immunologically active organ, provides a crucial physiological barrier against external pathogens and environmental stresses (14, 43). Rainbow trout are widely cultured throughout the world for commercial aquaculture. However, little is known about differences in immunity between WR_S and YR_S in a natural flowing water pond culture environment. Recently, a growing number of ncRNAs, including miRNAs, lncRNAs and circRNAs, have been shown to have vital regulatory roles in gene expression networks that influence numerous biological processes, especially immune regulation (20). To better understand the difference in innate immunity between WR_S and YR_S, we systematically compared the whole transcriptome of their skin, and thousands of dysregulated transcripts were identified, including 2448 mRNAs, 1630 lncRNAs, 22 circRNAs and 50 miRNAs. Furthermore, a set of potential mRNAs and ncRNAs involved in the immune regulation were selected to construct lncRNA-miRNA-mRNA and circRNA-miRNA-mRNA co-expression networks. The findings facilitate our understanding of the innate immune system of rainbow trout, and lay the foundation for further study of immune mechanisms and disease resistance breeding.

The RIG-I like receptor signaling pathway was significantly enriched in this study, indicating its importance in the skin defence system. Three members of the RLR family (*ddx58*, *ifih1* and *dhx58*) have been identified in mammals (44), but *ddx58* is limited to ancient fish such as grass carp (*Ctenopharyngodon idella*), common carp (*Cyprinus carpio*), zebrafish and goldfish (*Carassius auratus*). *ifih1* and *dhx58* are present in most common fish including rainbow trout (45, 46). Our current results are consistent with those of previous studies; *ifih1* and *dhx58* were identified, but *ddx58* was not. *ifih1* in teleost has been proved to play a key role in antiviral immunity, which senses the cluster signal of TLRs infected with RNA virus and initiates innate immune signaling cascades, thereby activating NF-κB- and IRF3/IRF7-mediated IFN response and establishing an antiviral state (40, 47). Rainbow trout *dhx58* acts as a positive regulator for IFN production through facilitating interaction between IFIH1 and RNA viruses (46, 48). The overexpression of *ifih1* can provide protection for Japanese flounder (*Paralichthys olivaceus*) against the invasion of infectious pancreatic necrosis virus and hirame rhabdovirus (49). In European sea bass (*Dicentrarchus labrax*), both *ifih1* and *dhx58* were upregulated upon infection with betanodaviruses (50). Similarly, overexpression of *trim25* severely reduced the transcription of viral genes and increased the expression of IFN-related molecules in orange spotted grouper (51). Consequently, upregulation of these genes in the RIG-I like receptor signaling pathway suggests that WR_S may possess stronger antiviral activity than YR_S in the natural flowing water pond culture environment. In the analysis of lncRNA-miRNA-mRNA network, it was found that 136 lncRNAs were identified to target *ifih1*, *dhx58* and *irf3* by binding to PC-5p-43254_34, PC-3p-28352_70 and bta-miR-11987_L-1R-1_1ss8TA. Among these three miRNAs, bta-miR-11987_L-1R-1_1ss8TA has been reported to be essential for the anti-cancer activity (52). Besides, lncRNAs like MSTRG.11484.2, MSTRG.32014.1 and MSTRG.29012.1 have a one-to-one regulatory relationship with *ifih1*, *dhx58* and *irf3*, respectively, suggesting they participate in the process of immune responses *via* RIG-I like receptor signaling pathway.

In addition to IFN response, inflammatory response is also a self-defense process that fights the pathogen invasion by eliminating harmful stimuli. Upregulated genes included one heat shock protein 90 (HSP90) family member (*hsp90a.1*) and two NLRs (*nlrp3* and *nlrc3*), all of which are involved in the immune response NLR signaling pathway that triggers inflammatory response *via* the activation caspase-1 and the release of cytokines (53). *nlrp3* is an important part of the inflammasome, and SGT1-HSP90 complexes work together to maintain its pre-activated stable state. When encountering irritation, *nlrp3* is separated from complexes and activated to induce caspase-1 through changes in *hsp90*, and it regulates the maturation of cytokines IL-1β and IL-18 to trigger inflammatory response (54–56). Anti-immunotoxic effects of *nlrp3* upon increased expression have been demonstrated in grass carp (57). Additionally, recombinant IL-1β can increase the number of leucocytes and phagocytes, and lysozyme activity in rainbow trout (58). Therefore, upregulation of *nlrp3* may contribute to improving defences in WR_S together with *sgt1-hsp90*. Furthermore, previous research showed that expression of *hsp90* was upregulated under heat stress in rainbow trout (39, 59), which implies that WR_S may have better immune defences than YR_S, resulting in greater capacity for dealing with heat stress. However, *nlrc3* was also upregulated in WR_S, which has a negative effect on inflammatory response (60). Although inflammation is a protective immune response against external environmental stresses, excessive inflammation can cause damage to the body, and it can be stimulated or suppressed by heat shock proteins *via* changes in other molecules (2, 61). In humans (*Homo sapiens*), expression levels of *nlrc3* were higher in healthy tissues than in cancerous tissues (62). These results indicate that excessive inflammatory response may be inhibited directly or indirectly through *hsp90* genes to protect the body of WR_S.

Phagolysosomes rely on a strongly acidic environment and associated complex immune mechanisms to play an important role in eliminating pathogens, and *V-ATPases* are essential for the formation of the highly acidic environment and bactericides such as H_2_O_2_ in phagolysosomes (63). In the present study, *atp6v1e1* was downregulated in WR_S, suggesting that the ability to remove and digest ingested pathogens in phagolysosomes may be diminished (64). Besides, we also identified some adaptive immune molecules in the phagosome pathway, including *tap1*, *tap2* and *mhci*, all of which were upregulated in WR_S. Once pathogens are phagocytosed by dendritic cells, peptides from these antigens are carried from the cytosol to the endoplasmic reticulum (ER) by *tap1* and *tap2*, which bind to *mhci* to form the peptide-MHCI complex, and this presents the antigens to cytotoxic CD8^+^ T-cells (CTLs) that kill MHCI-matched infected cells in teleost fish (65–67). Knockout and mutation of *tap1 *and *tap2* significantly reduce the number of MHCI complexes, which results in autoimmune disorder and susceptibility to infections (66, 68), indicating that timely shipment of antigens to CTLs *via* TAPs is crucial in preserving the normal functions of the immune system. Previous studies on infected skin of orange spotted grouper (12) and rabbitfish (13) showed that *tap1*, *tap2*, *mhci* and *cd209* were upregulated in infected samples, suggesting that more antigens were displayed to CTLs through increased expression of *taps* in the defence against pathogens. In zebrafish, *cd209* blockade inhibits T cell activation (69). It is therefore interesting that *cd209* was also upregulated in WR_S in the present work, which indicates that CTLs are more active. Taken together, upregulation of these genes suggests that innate and adaptive immune responses in phagosome may be a major part of the immune defences in YR_S and WR_S, respectively. From the result of the circRNA-miRNA-mRNA network, we found that circRNA5279 and circRNA5277 were co-expressed with *tap2* through competitively binding with oni-mir-124a-2-p5_1ss13GA. Mir-124a was identified as a key factor in regulating T cell activation and differentiation (70, 71). A recent report showed that reduced level of mir-124a is associated with increased proinflammatory mediator expression in mice (*Mus musculus*) (72). These results implied that circRNA5279 and circRNA5277 may play an important role in skin immunity of rainbow trout.

In addition to being the largest immune organ, fish skin is also considered a metabolically active tissue (4). In recent years, numerous metabolic biomarkers that regulate immune responses have been identified in fish, demonstrating that the immune response is closely related to metabolism. In the present study, many DEmRNAs were enriched in metabolic pathways, indicating that differential expression of these genes may be responsible for differences in immunity between WR_S and YR_S. Besides, 88 lncRNAs were localized in the metabolism-related pathways and competitively targeted mmu-mir-6236-p3_1, mmu-mir-6236-p3_2 and mmu-mir-6236-p5_1, which regulated *gstp1*. We found that expression of *gstp1* was upregulated in YR_S. GSTs are related to the metabolism of xenobiotics by cytochrome P450, and to glutathione metabolism. Previous studies found that exposure of zebrafish and Nile tilapia (*Oreochromis niloticus*) to certain concentrations of endosulfan and metals (Cd, Cu, Cr, Pb and Zn) can result in the upregulation of GSTs (73, 74), which may indicate that YR_S are more susceptible to xenobiotics in the natural pond culture environment. Moreover, many xenobiotics without inherent immunotoxicity can be converted into highly immunotoxic metabolites by cytochrome P450 enzymes, and their activity can be downregulated by inflammatory cytokines in fish (75). Thus, upregulation of pro-inflammatory genes in WR_S suggests that the metabolic rate of xenobiotics may be slowed.

Upregulation of *nampt*, *naprt* and *cd38* were also detected in WR_S, and these genes function in nicotinate and nicotinamide metabolism, as well as NAD biosynthesis. Furthermore, two GO terms were assigned to NAD+ADP-ribosyltransferase activity (in the molecular function category) and NAD biosynthetic process (in the biological processes category), the former of which was the most significantly enriched among all GO terms. NAMPT and NAPRT are rate-limiting enzymes that catalyse the synthesis of NAD from nicotinic acid and nicotinamide (the two main precursors), and their increased activity can accelerate the rate of NAD synthesis (76, 77). Studies have shown that enhancing NAD synthesis can hinder the spread of various diseases and alleviate adverse inflammatory response (78, 79). Additionally, NADH can reduce O_2_ to H_2_O_2_, and the activity of cytochrome P450 enzymes is suppressed by nicotinamide (71, 80). Hence, the upregulation of *nampt* and *naprt* plays an essential role in protecting the body against environmental stress in WR_S. *cd38* is a major consumer of NAD, which can be degraded into nicotinamide, and lacking *cd38* displays an increase in NAD content (78). In mice, inhibition of *nampt* results in a decrease in *cd38* activity (81). Therefore, upregulation of *cd38* may be increased along with *nampt* and *naprt* in WR_S.

Melanocytes, melanin-producing cells located in the superficial layers of skin, are very important components of the innate and adaptive immune responses of fish skin. In the process of skin melanisation caused by black melanin, numerous toxic intermediate compounds are generated, including quinones (dopaquinone, indolequinones and semiquinones) and various ROS with strong antimicrobial properties (82). The stratum corneum can also be acidified in black skin when melanosomes of melanocytes are transported to keratinocytes on the outside of the epidermis, where the acidic environment is conducive to antimicrobial function (83). Additionally, pattern recognition receptors (PRRs) composed of RLRs, NLRs and TLRs are expressed on melanocytes, which can induce the production of type I IFNs, cytokines (IL-1β, IL6 and TNF-a) and chemokines to stimulate immunity (84). Moreover, melanomacrophages, T cells and MHC class II cells are present in the black spots of salmon, and melanocytes function similarly to antigen-presenting cells in human (82, 85). Individuals with white skin were found to be more susceptible to infections than those with black skin (86), indicating that melanocytes may play important roles in the battle against pathogenic microorganisms *via* innate and adaptive immune responses and maintenance of skin homeostasis. In addition to melanocytes, carotenoids contribute to a more robust immune response by protecting phagocytic cells from autooxidative damage, stimulating effector T-cell function, and enhancing T- and B-lymphocyte proliferation (87). Xanthophores contain lots of carotenoids, suggesting that the number of xanthophores is positively correlated with immunity. Thus, the large number of melanocytes and xanthophores present in the skin of WR_S may strengthen the skin mucosal immune system. Additionally, the microbiome inhabiting fish skin mucus is crucial for immune function in the mucosal epithelia. Several studies have reported differences in disease-resistant and susceptible fish that are correlated with differences in the skin microbiome (6, 88–90). Furthermore, 16S rRNA analysis revealed differences in microbiome composition between melanin-deposited and pseudo-albino skin in fatfish (*Pleuronectiformes*) (91). Similar results were also found in frogs (*Agalychnis callidryas*) and humans, showing that the skin microbiome composition varies with skin colour (92, 93). Therefore, the skin immune status of WR_S and YR_S may be affected by differences in the microbiome, but further studies are needed to confirm this.

## Conclusion

In the present study, the skin expression profiles of mRNAs, lncRNAs, circRNAs and miRNAs were detected by whole transcriptome sequencing to explore differences in immunity between WR_S and YR_S in a natural flowing water pond culture environment. After differential expression analysis, a great number of key immune-related DEmRNAs and DEncRNAs were identified, and cluster analysis showed that most of the DEmRNAs were implicated in immune and metabolism-related pathways. In addition, two co-expression networks (lncRNA-miRNA-mRNA and circRNA-miRNA-mRNA) were constructed to better understand the regulatory relationships of these mRNAs and ncRNAs. These results broaden our understanding of the innate immune system between these two phenotypically distinct variants, and provide a basis for further study of immune mechanisms and resistance breeding in rainbow trout.

## Data Availability Statement

The datasets presented in this study can be found in online repositories. The names of the repository/repositories and accession number(s) can be found below: https://www.ncbi.nlm.nih.gov/geo/, accession ID: GSE153997.

## Ethics Statement

The studies involving human participants were reviewed and approved by Gansu Agricultural University. The patients/participants provided their written informed consent to participate in this study. The animal study was reviewed and approved by Gansu Agricultural University.

## Author Contributions

SW wrote the manuscript. JH designed the experiment. YL and ZL conducted data analysis. LZ collected samples. All authors contributed to the article and approved the submitted version.

## Funding

This work was financially supported by the National Natural Science Foundation of China (Grant No. 31760755), the Fuxi Youth Talent Training Program of Gansu Agricultural University (Grant No. Gaufx-02Y08).

## Conflict of Interest

The authors declare that the research was conducted in the absence of any commercial or financial relationships that could be construed as a potential conflict of interest.

## Publisher’s Note

All claims expressed in this article are solely those of the authors and do not necessarily represent those of their affiliated organizations, or those of the publisher, the editors and the reviewers. Any product that may be evaluated in this article, or claim that may be made by its manufacturer, is not guaranteed or endorsed by the publisher.

## Abbreviations

WR_S, wild-type rainbow trout; YR_S, yellow mutant rainbow trout; DEmRNAs, differentially expressed mRNAs; DElncRNAs, differentially expressed lncRNAs; DEcircRNAs, differentially expressed circRNAs; DEmiRNAs, differentially expressed miRNAs; ncRNAs, non-coding RNAs; *tlrs*, Toll-like receptors; *nlrs*, NOD-like receptors; ceRNA, competing endogenous RNA; MREs, common miRNA response elements; RIN, RNA integrity number; CPC, Coding Potential Calculator; CNCI, Coding-Non-Coding-Index; GO, Gene Ontology; KEGG, Kyoto Encyclopedia of Genes and Genomes; qRT-PCR; quantitative reverse transcription real-time PCR; FPKM, fragments per kilobase of transcripts per million fragments mapped; ORF, open reading frame; RLR, RIG-I-like receptor; PPI, protein-protein interaction; CTLs, CD8^+^ T-cells; ROS, reactive oxygen species; PRRs, pattern recognition receptors.
