# Supplementary figures and images for: Integrated Analysis of lncRNA and circRNA Mediated ceRNA Regulatory Networks in Skin Reveals Innate Immunity Differences Between Wild-Type and Yellow Mutant Rainbow Trout (Oncorhynchus mykiss)
Source: Front Immunol. 2022 May 17;13:802731. doi: 10.3389/fimmu.2022.802731 (PMC9152293; doi:10.3389/fimmu.2022.802731)

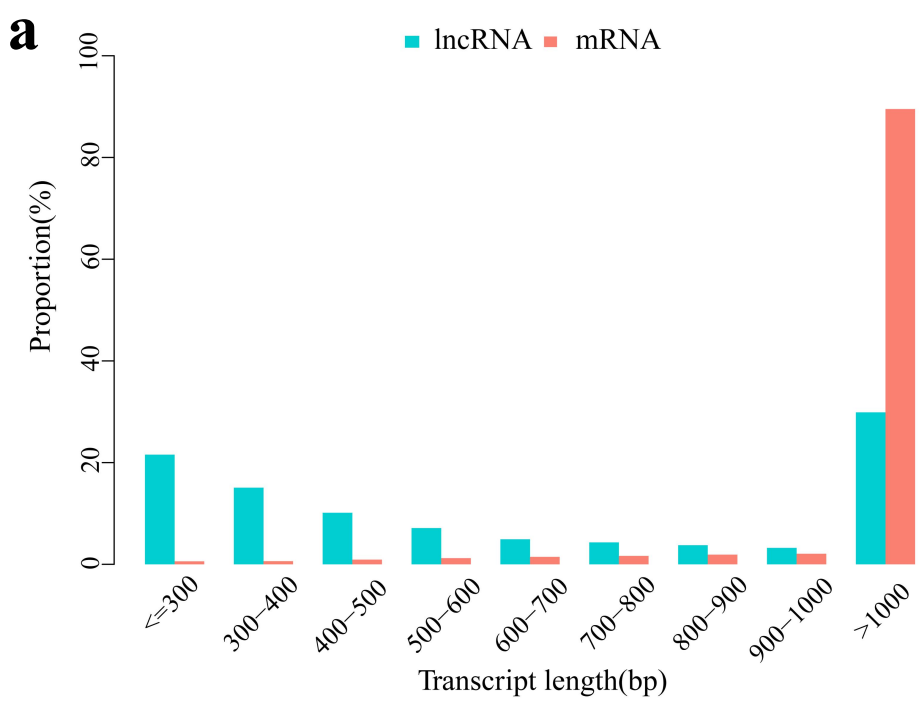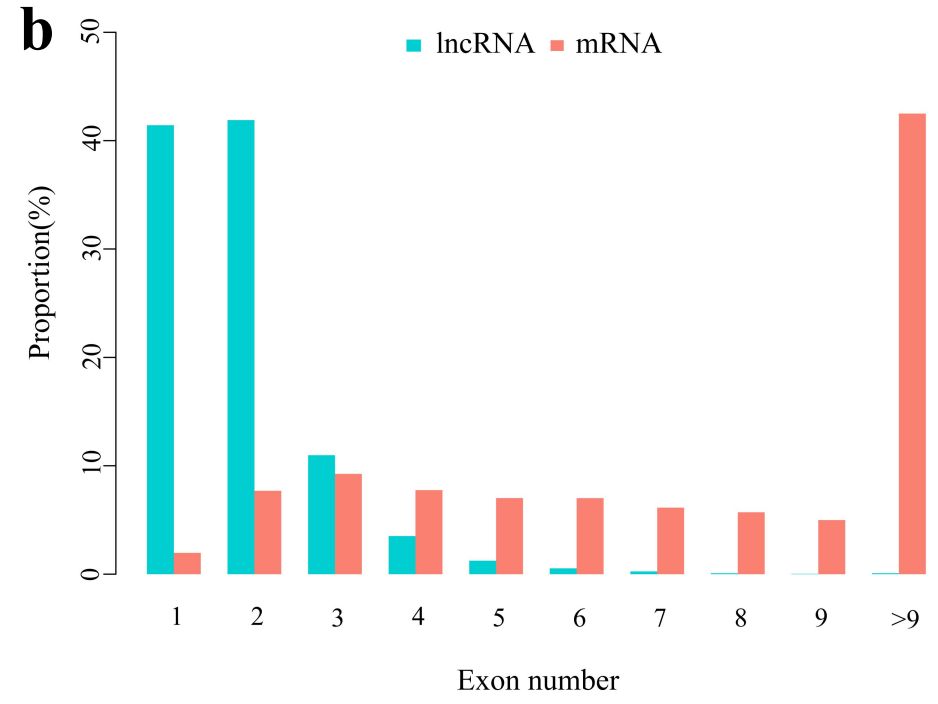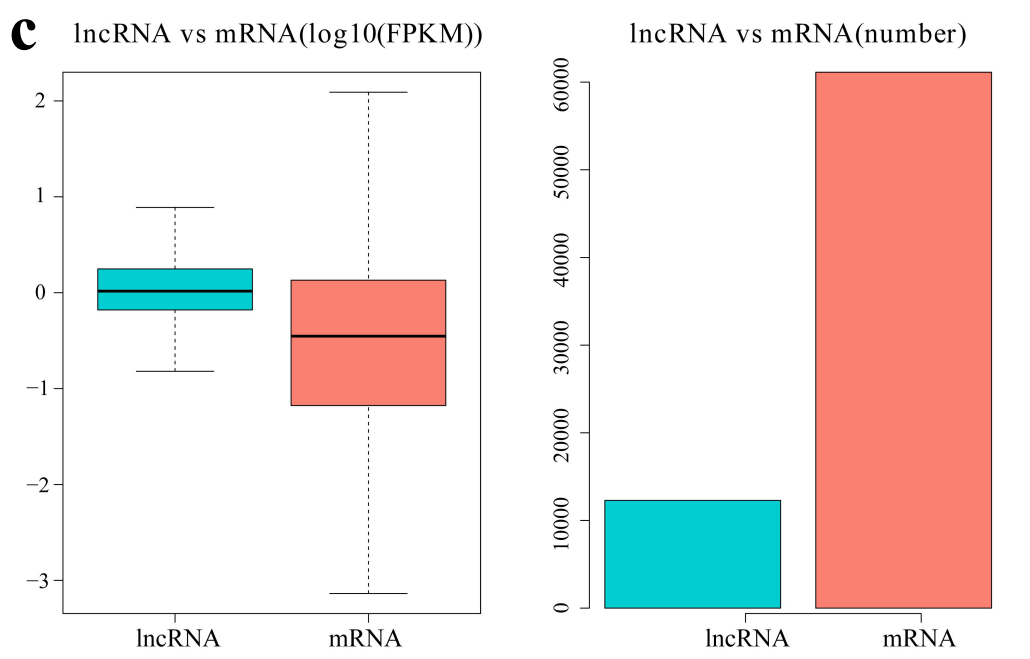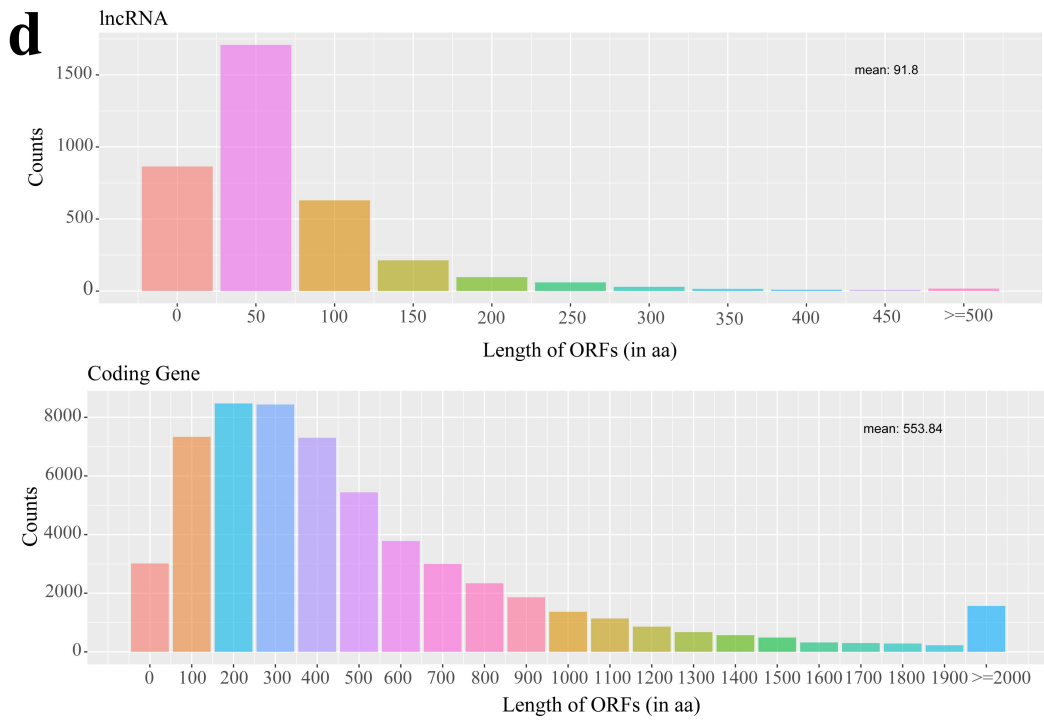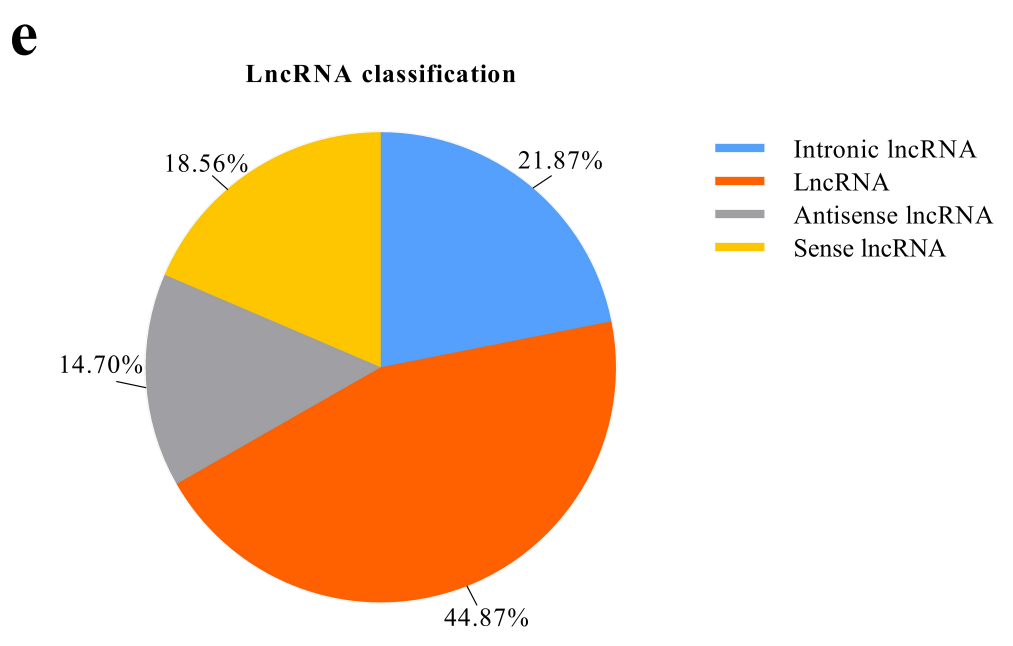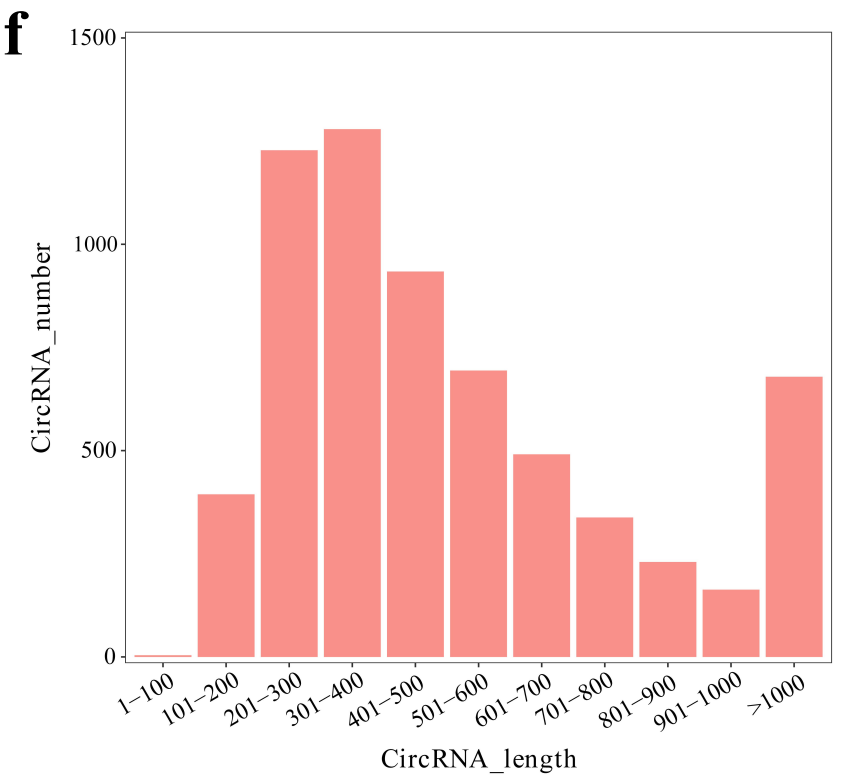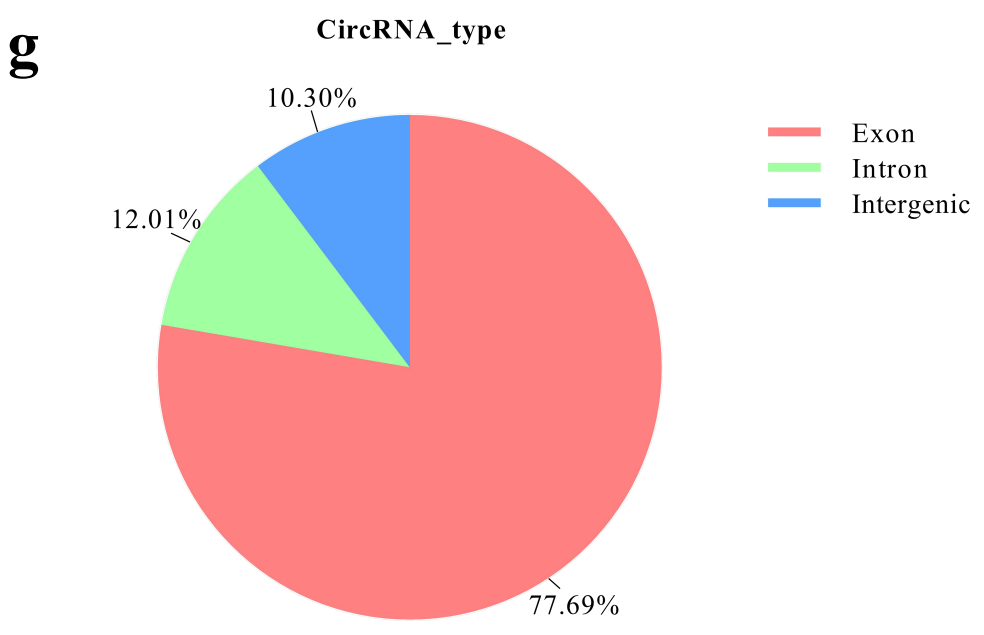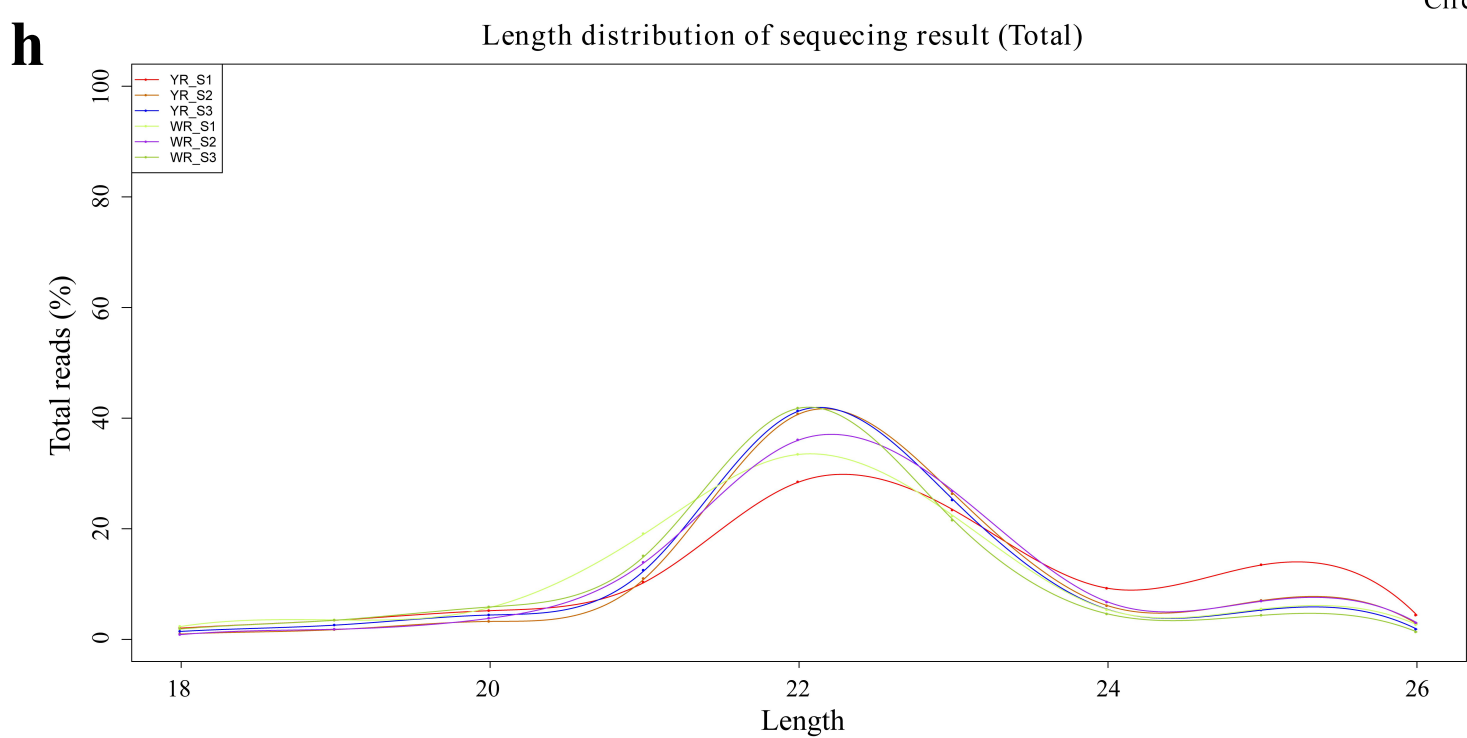

Supplement: Supplementary file 1 [file DataSheet_1.pdf]
